# Bibliometric analysis of global research trends on pyroptosis in lung disease

**DOI:** 10.3389/fimmu.2022.978552

**Published:** 2022-09-13

**Authors:** Wei Mo, Quanfu Li, Huanping Zhou, Xuan Shi, Hao Yang, Zhuoran Xiao, Juan Wei, Xin Lv

**Affiliations:** ^1^ Graduate School, Wannan Medical College, Wuhu, China; ^2^ Department of Anesthesiology, Shanghai Pulmonary Hospital, School of Medicine, Tongji University, Shanghai, China

**Keywords:** pyroptosis, lung, bibliometric analysis, VOSviewer, CiteSpace

## Abstract

**Background:**

Pyroptosis is a lytic pro-inflammatory programmed cell death mode that depends on caspase, inflammasome, and Gasdermin D (GSDMD). A growing number of studies have shown that pyroptosis is closely related to the pathophysiological mechanism of lung. The purpose of this study is to analyze the literature from Science Citation Index Expanded (SCI-expanded) of Web of Science Core Collection (WoSCC) and visualize the current trends and hotspots in the research of pyroptosis in lung disease.

**Methods:**

On February 20, 2022, we retrieved all articles on pyroptosis in lung disease from SCI-expanded of WoSCC. Original articles and reviews published in English from 2007 to 2021 were included in the analysis. VOSviewer 1.6.17 and CiteSpace 5.8.R2 were used to analyze the retrieved data and visualize the results.

**Result:**

1798 qualified original articles and reviews on pyroptosis in lung disease were included in the bibliometric analysis. So far, the research in this field is still in a period of growth, and the number of global publications has increased yearly. Among the 66 countries that have published relevant articles, China ranked first in the number of publications, and the USA ranked first in the number of cited articles. Holian,A. was the author with the largest number of articles, including 21 published. The University of California System in the USA was the organization with the largest number of articles, totaling 55. Frontiers in Immunology was the journal with the most publications in pyroptosis. After bibliometric analysis, the frequently used keywords are: “NOD-like receptor3 (NLRP3) inflammasome”, “inflammation”, “oxidative stress”, and “acute lung injury (ALI)”.

**Conclusion:**

The research on pyroptosis in lung disease is in its growth stage. The information released in this article may help researchers better understand the hotspots and developmental trends in this field, the cooperation network information of authors, countries, and institutions, and the citation correlation between articles. With the in-depth study of the mechanism of pyroptosis, the focus has shifted to increasing research on the connections and influences of different diseases. So far, increasing attention has been paid to the research field of the relationship between ALI and pyroptosis related to COVID-19.

## Introduction

The term “pyroptosis” was first proposed in D’Souza’s article (1). Pyroptosis is a lytic and regulatory programmed cell death depending on caspase and inflammasomes (2). One of the main characteristics of pyroptosis is its dependence on caspase. Caspase is a cysteine protease (3), which plays a pivotal role not only in the process of pyroptosis but also in apoptosis, another programmed cell death mode. Pyroptosis and apoptosis have some similar characteristics, such as caspase dependence, chromatin concentration, and DNA breakage (2). On the other hand, as gasdermin D (GSDMD) is responsible for pore formation on the plasma membrane, many pro-inflammatory mediators, such as interleukin-1β(IL-1β) and IL-18, are actively secreted through the membrane pore of the cell when the cell membrane is destroyed, which affects the intracellular osmotic pressure and facilitates penetration of water into cells, resulting in cell swelling and dissolution, thus inducing cell pyroptosis. From then on, the cascading reaction of inflammation begins (4). More lung-related diseases have emerged such as lung cancer, forming a crux in the malignancy landscape (5), and acute respiratory distress syndrome (ARDS) in observational studies from across the world, which the mortality of patients with it remains high (6–8). Despite the progress in the research of pyroptosis in lung disease in recent years, many issues tarry to be explored.

Bibliometric analysis is a new framework of research analysis of impact and trend, which is gaining increased popularity (9). Bibliometrics is a research method of quantitative analysis by using literature metrology characteristics (10) and visual display of the research results of journals and disciplines in a certain field based on various statistical analysis software and methods. Bibliometric methods mainly include citation analysis. Meanwhile, a large number of cited articles are considered to be the core and spotlight of the research (11). Research on pyroptosis in lung disease will also be a research hotspot for a long time. Therefore, it is particularly important to understand the current research hotspots and development trends in this field, which is helpful to explore diseases in lung-related fields and solve clinical problems.

Firstly, we briefly introduce the application methods of this study, then show the trend of publications regarding annual growth, most influential institutions and journals in pyroptosis in lung disease, where the collaborations between the most productive countries and authors are visualized using VOSviewer, CiteSpace and the bibliometric package of the R language.

## Materials and methods

### Data sources and search strategies

**Figure 1:**
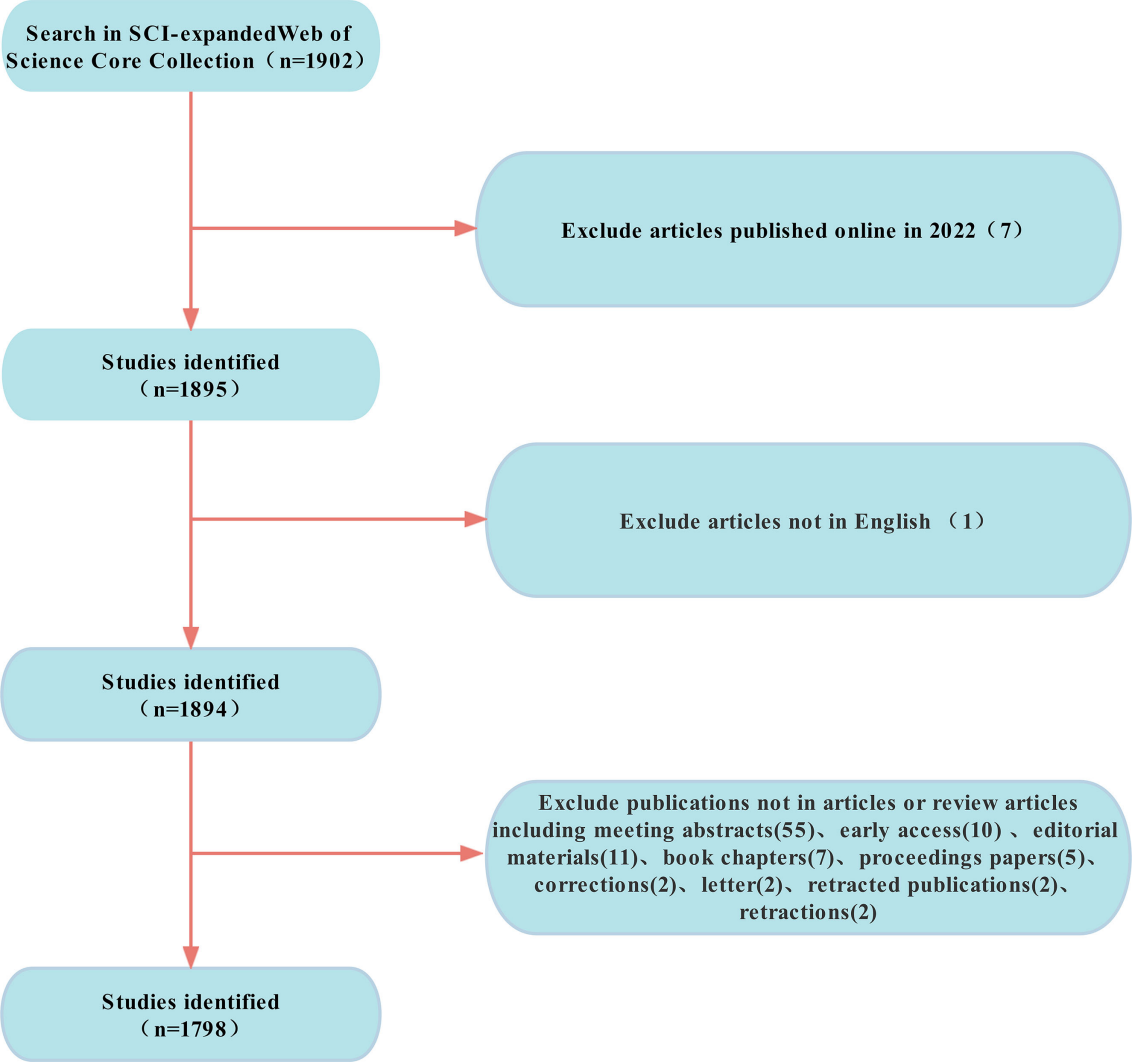
Flowchart of the sifting process.

We used WoSCC in this study, knowing that it is the most frequently accepted database developed by Thomson Science for scientific or bibliometric studies and contains comprehensive citation index records that include numerous influential and high-quality journals (12). Moreover, a study has illustrated that the Web of Science has better accuracy in document type labels than any other database such as Scopus. All articles and reviews published online on pyroptosis in lung disease were retrieved from SCI-expanded of the WoSCC between 2007 and 2021. The MeSH term of ‘pyroptosis’ is like that in the previous literature (13). The search strategy that we used in the WoSCC database is as follows: TS= (pyroptosis OR pyroptosic OR inflammasome OR pyroptosome) AND TS= (lung). Two reviewers (WM and ZRX) searched the field separately, comparing and sorting each other, and read the abstract and, if necessary, the whole text, resulting in a final compliance of 90%, showing substantial accordance (14). Subsequently, we excluded the online publication time of 2022, non-English, and limited the publication type to reviews and original articles. Finally, a total of 1798 original articles and reviews met the criteria for inclusion in the analysis, and further visual analyses were carried out **(** **Figure 1** **)**.

### Bibliometric analysis

Through the two kinds of indicators of objective bibliometrics and evaluation bibliometrics, this paper made a visual analysis of the research results in a certain field. Positive bibliometrics refers to the number of documents, the number of citations, citation analysis, etc. Evaluative bibliometrics is a subfield of quantitative science and technology research that constructs research performance indicators (15). Evaluative bibliometrics refers to the quantitative evaluation of the contributions of countries, authors, magazines, and institutions in this field, as well as their quantitative indicators, such as the H-index and impact factor (IF). This kind of analysis can determine the articles that affect the history of a certain field, current research hotspots, and future development trends (16). We visualized the results according to the annual number of articles, countries, organizations, authors, citations, and other related aspects. We used the data analysis function of the WoSCC database. Before data analysis, EndnoteTM 20 and the Bibliometrix Package based on the R4.1.1 were used to store, count, and clean data. The H-index and IF were both included in the analysis as important indication of the research’s scientific (17). Then, we use the following software for bibliometric analysis.

VOSviewer v.1.6.17 is a free JAVA-based software developed by Nees Jan van Eck and Ludo Waltman in 2009, analyzing a large number of literature data in an easy-to-interpret way and displaying it in the form of a map (18). This software operates based on co-occurrence matrix, and its clustering algorithm is based on association strength (19). In other words, the similarity of nodes, shown by using the software function of distance visualization. The closer the distance of nodes with greater similarity is, the further the distance of nodes with smaller similarity is (20). VOSviewer was used to make further visualizations of the co-authorship of the authors, institutions, countries, and the co-occurrence of keywords. Among 10504 authors, the minimum number of articles published by an author was set to 6. Countries that met the requirements of posting more than 5 articles are shown in the visual graph. Although 81 institutions met the threshold of 10 articles. At least 20 of the 6412 keywords appeared, and a total of 144 keywords were included in the co-occurrence analysis of the software and the total intensity of co-occurrence bonds to keywords was also derived. Furthermore, we uniformed ‘IL-1β, IL-1α, interleukin-1 and interleukin-1β’ to ‘IL-1’. In the visual graph, the node size represents the number of articles, the node color represents different clusters, the connection between nodes represents the association strength of the two nodes, and the distance represents the association degree.

The bibliometric package of R language v. 4.1.1 was used for data cleaning, such as the writing format, i.e. case unification and synonym merging. In addition, R language was also used to make the radar map in this study.

CiteSpace v.5.8. R2, also a Java-based citation visualization software, presuming an experimental platform for investigating new ideas and comparing existing methods (21), visualizes the research results of an area by drawing the literature co-citation network map, to understand the knowledge domain, research frontier, and development trend, and predict its future research progress (22). CiteSpace’s cluster analysis function classifies and arranges keywords and references and shows the key contents of pyroptosis in lung disease research. Using this software, we not only made the dual-map overlay of articles citing but also made the timeline map of literature keywords in this field from 2007 to 2021 to show the changing trend of keywords in different periods and the relationship between clusters. The dual-map overlay is a new method of visual publication portfolio analysis, to depict the subject distribution of academic journals, which includes analyzing, and comparing the combined characteristics of publications (23, 24). In addition, the bursts of keywords are often used to intuitively understand the research hotspots and evolution process and forecast new research trends of the area of interest (25).

## Results

### The overall trend of the number of documents issued

**Figure 2:**
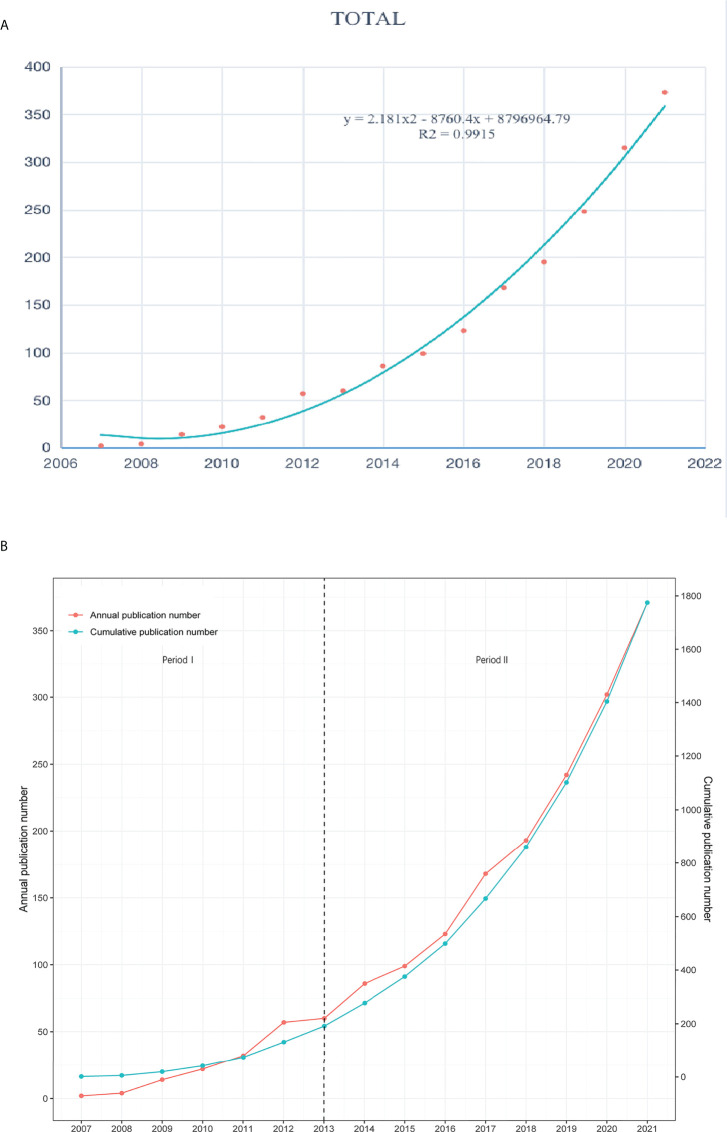
Global publications of research results on pyroptosis. **(A)** The figure-fitting curve of global document volume changing with the years. **(B)** Growth model of document volume over time.

It has been 20 years since pyroptosis was proposed by American scholars in 2001, while research about pyroptosis in lung disease research began to develop in 2007. **Figure 2A** showed the trend fitting curve of the total amount of literature in this field with years. According to the figure-fitting curve analysis, the number of annual publications on pyroptosis in lung disease showed an upward trend (R^2^ = 0.9915). There are many kinds of general literature growth models, but at present, the nonlinear fitting curve model is more widely used to judge the development status of research in a certain field and predict the future literature research direction. The nonlinear fitting curve model has a more ideal prediction effect and fitting accuracy (26). The S-curve model generally divides the literature growth trend into four periods: initial stage, growth period, stable period, and theoretical improvement period. **Figure 2B** depicted the growth trend of the annual publications issued in pyroptosis in lung disease, which could be divided into two stages. The theory of Period I, from 2007 to 2013, was not completed, which indicates this topic had just been concerned by scholars. In period II from 2013 to 2021, also known as the growth period, the research in this field made remarkable progress over years, and more scholars paid increasing attention to this field and achieved more scientific research achievements, thus promoting the further development of exploration in this field.

### Country/region distribution

**Figure 3:**
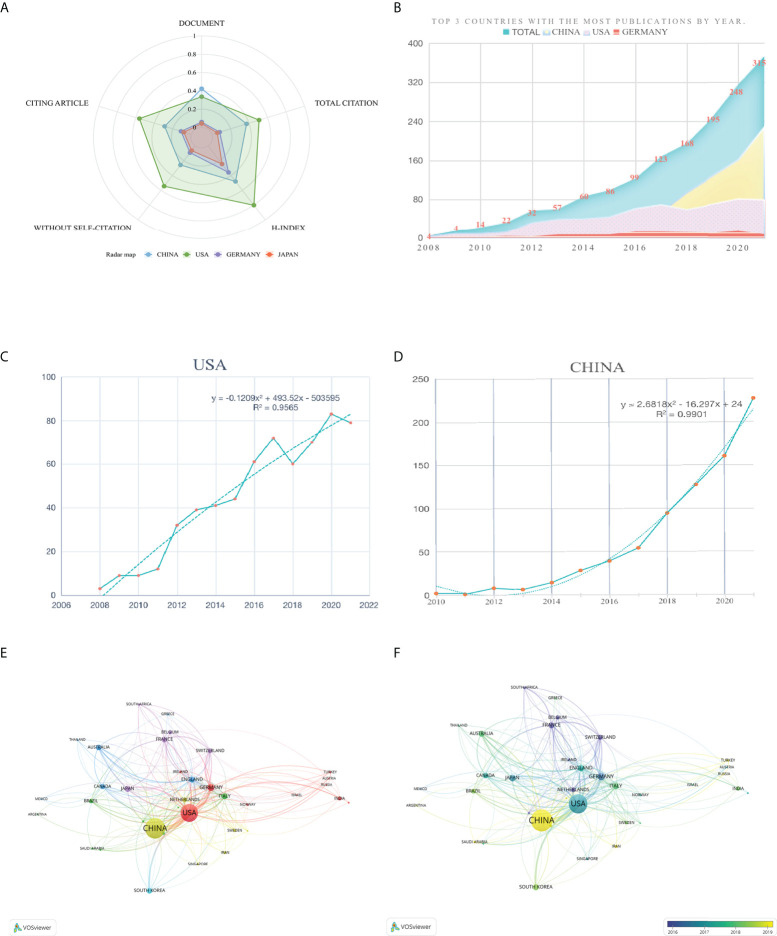
National trends in the number of papers on pyroptosis in lung disease over time. **(A)** Radar chart of relevant evaluation of the top four countries in the volume of documents issued. **(B)** Comparison of the top three countries in the number of documents issued. **(C)** The fitting curve of the variation law of the number of documents issued in the USA with years. **(D)** The fitting curve of the variation law of the number of documents issued in China with years. **(E)** Network visualization showing the relationship between countries. **(F)** Time-related country network relationship map in this region.

In 2021, pyroptosis in lung disease was studied in 66 countries and regions. The number of literatures increased yearly, from 4 in 2008 to 373 in 2021 **(** **Table 1** **)**. The USA was not only the country with the largest number of citations but also the country with the top cited. The number of citations in the USA not only was twice that of Chinese articles but also there was a greater gap compared with the number of citations in other countries **(** **Figure 3A** **)**. Contemporary, China had the largest number of documents (760 publications, 42.27%), followed by the USA (604 publications, 33.59%), Germany (106 publications, 5.89%) and Japan (77 publications, 4.28%). From 2012, the annual number of publications in the USA and Germany kept counterbalanced, while in China, the quantity of achievements on pyroptosis in lung disease had increased significantly to the top-one country in recent years**(** **Figure 3B** **)**. The annual number of publications of USA and China was considerably correlated with the publication year, and the correlation coefficient R^2^ reached 0.9565 and 0.9901 respectively **(** **Figure 3C, D** **)**. In 2021, China accounted for 60.86% of the tally of articles in this research world, exceeding the total number of articles published by other countries in the world **(** **Figure 3D** **)**. The visualization produced by VOSviwer software depicted the cooperation among countries **(** **Figure 3E** **)**. Among them, China had cooperated with 30 countries and the USA had collaborated with 45 countries. The cooperation between China and USA was largest, with a total of 99 publications, of which publications in the past five years accounted for 69.7% of the tally of articles **(** **Figure 3F** **)**. Since 2007, the USA, Germany, France, and other countries had started to focus on research in this region. However, the research on this zone in China began to bloom only in 2010. Many countries did not appear in **Figure 3E**, indicating that their ties with other countries are not proximate enough. Only by strengthening collaboration among countries can we break down scientific research barriers and promote the sound development of scientific research.

**Table 1:** Top10 countries with the most documents.

Rank	Country	No. of documents	Total citations	Citing articles	Average citations per item	H-index
1	CHINA	760	14653	11290	19.25	51
2	USA	604	29163	21848	48.28	86
3	GERMANY	106	4785	4345	45.14	38
4	JAPAN	77	3312	3162	43.01	26
5	ENGLAND	70	2456	2296	35.09	25
6	ITALY	70	2086	1926	29.8	23
7	FRANCE	64	3561	3122	55.64	28
8	SOUTH KOREA	64	1083	997	83.31	12
9	AUSTRALIA	62	2187	2039	35.27	26
10	CANADA	56	1860	1737	33.21	24

### Authors and co-cited authors

Since 2007, a total of 10504 authors had participated in the study of pyroptosis in lung disease, publishing a total of 1798 articles. The top ten core authors and their number of publications, the total citations, and the H-index were shown in **Table 2**. The top 10 authors published 150 articles and enjoyed a total of 12034 citations, accounting for 8.34% and 21.08% respective total number. Holian A from the University of Montana was the most productive author, enjoying 21 achievements and 1009 citations. The author with the most citations in the table was Couillin I from the University of Orleans and Centre National de la Recherche Scientifique in France enjoying 2051 citations. Additionally, the second most cited author is Ryffel B from the University of Orleans in France, enjoying 1904 citations. Brooke Mosman, the most co-cited author of pyroptosis in lung, was not among the top 10 authors, enjoying 2252 times. His article “innate immune activation through NALP3 inflammasome sensing of asbestos and silica” (27) issued in May 2008 was cited 1821 times, which was the most cited article in this research area. The above data showed that although the number of published articles by Brooke Mosman and his team shrank is not large enough, the quality of their articles was relatively high, which had conspicuously pushed forward the progress in this sphere. To sum up, not only the number of articles but also the quality and issuing time of the productions need to be considered in the evaluation of productive authors.

**Table 2:** Top 10 authors with the most documents.

Rank	Author	Institution	Country	No. of articles	Total Citations	H-index	Total link strength
1	Holian, A	University of Montana	USA	21	1009	15	42
2	Ryffel,B	University of Orleans	France	18	1904	20	36
3	Xia,T	University of California System	USA	17	1223	17	102
3	Wang,X	University of California System	USA	17	1224	17	102
5	Sun,B.B.	University of California System	USA	14	941	11	86
5	Couillin,I	University of Orleans and Centre National de la Recherche Scientifique	France	14	2051	15	39
7	Chang,C.H	University of California System	USA	13	1014	13	90
8	Liao,Y.P.	University of California System	USA	12	917	12	82
8	Choi,A.M.K.	New York Presbyterian Hospital	USA	12	885	11	11
8	Li,R.B.	University of California LosAngeles	USA	12	866	10	76

Among the top 10 authors, half of them is from the University of California systems, Xia T with 17 publications, Wang X with 15 publications, Sun B.B. with 14 publications, Chang C.H 13 with publications and Liao Y.P. with 12 publications, respectively. They had an inseparable relationship and have plenty of associations. Since 2013, certain high-impact achievements had been consummated by their cooperation, which mainly focalized on the mechanism of the interaction between nanomaterials or carbon nanotubes and pyroptosis in lung diseases (28). VOSviewer software was used to portray the cooperation between authors in this sphere. The node size, in the network visualization diagram, grown as the number of contributions of the author increases. The larger the node was, the more plenty of the author’s achievements were. The thickness of this line displayed also as the total link strength in **Table 2**, representing the strength of associations between the two authors. We stipulated that the number of articles issued by the authors included in the statistical analysis should not be smaller than 6, and finally only 98 authors reached the threshold.

**Figure 4:**
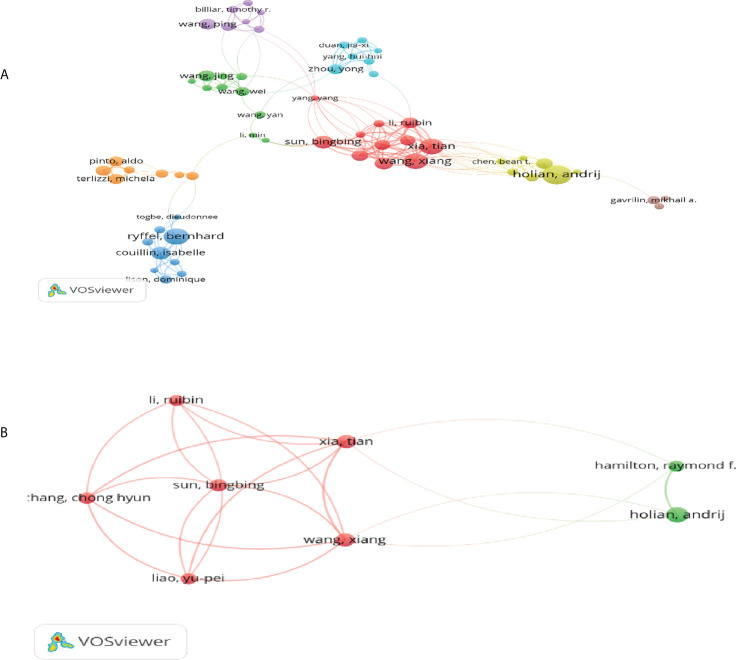
Visual analysis of the co-authorship analysis on pyroptosis in lung disease research. **(A)** Co-authorship analysis of the top 10 authors in term of the number of papers published in this field. **(B)** Visual network of authors publishing articles in this field and their relationships.

Through the visual map, we could not only understand the differences in the number of documents that they had but obtained the distinctness in the collaborative relationship among the authors. The software automatically divided all authors into different clusters. The cooperative relationship between authors in the same cluster was closer than that between different clusters. It could be the catch sight that the authors belonging to red clustering cooperated densely as well as contributed the immensely outstanding scientific research achievements in this region **(** **Figure 4A** **)**. Couillin I and Ryffel B had worked together but had less concurrence with the other top 10 writers. As a result, they did not appear in the top 10 authors’ partnership network visualization. Metaphorically speaking, Wang X and Xia T were like a bridge connecting the other top 10 authors who cooperated with each author as shown in **Figure 4B**. Xia T and Wang X once collaborated with Holian A and Ryffel B. Afterwards, Xia T, Wang X and other colleagues of the same organization had been conducting research and knowledge exploration in this area and published a multitude of invaluable investigation results. Cooperation was always an important prerequisite for scientific exploration progress.

### Institutions

1907 institutions, systematically, had launched articles on the theme of pyroptosis in lung disease. More than half of the top 10 institutions in the number of publications came from six affiliations in China, followed by the USA (2), Japan (1), and France (1). The affiliation that ranked first in terms of the number of documents and H-index was the University of California system from the USA (enjoying 55 documents, 2049 citations and 25 H-index). Shanghai Jiaotong University (enjoying 47 papers and 945 citations) ranked second, and the Central South University (enjoying 45 papers and 832 citations) ranked third **(** **Table 3** **)**. Of the 55 articles published by California institutions, 19 achievements (34.55%) came from the contributions of the top 10 authors. Scientific research had no boundaries, and nothing was more exceptionally indispensable than aggrandizing scientific research forward movement together.

**Table 3:** Top 10 institutions with the most documents.

Rank	Institution	Country	No. of articles	Total Citations	H-Index
1	University of California System	USA	55	2049	25
2	Shanghai Jiaotong University	China	47	945	20
3	Central South University	China	45	832	18
4	Centre National de la Recherche Scientifique	France	38	2534	22
5	Institut National de la Santé et de la Recherche Médicale	France	38	2107	20
6	Chinese Academy of Sciences	China	37	1468	17
7	Pennsylvania Commonwealth System of Higher Education	USA	36	1618	24
8	Fudan University	China	34	838	14
9	Huazhong University of Science Technology	China	33	1074	14
10	Chinese Academy of Medical Sciences Peking Union Medical College	China	32	385	9

### Journals

**Figure 5:**
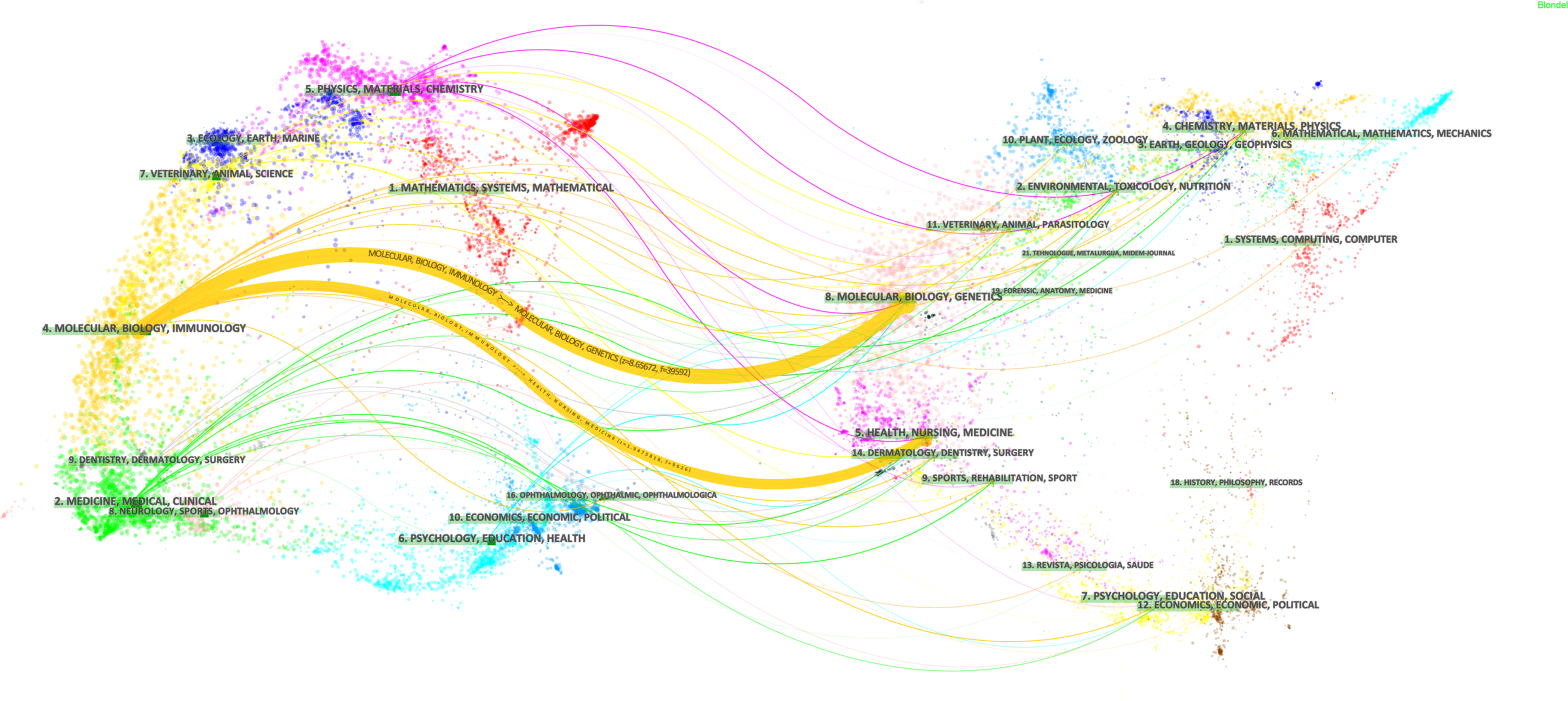
The dual-map overlay of citing of citation relationship of articles on pyroptosis in lung disease. The citing journal is on the left, and the cited journal is on the right. The citation relationship is showed by the colored path.

Altogether 542 journals published 1798 papers concerning pyroptosis in lung disease. The top 10 fruitful sources were listed in **Table 4**. The published 400 papers in this field accounted for approximately 22.25% of the total, but their IF did not outstrip 10, demonstrating that the quality of many studies was not high. Frontiers in Immunology was the most prolific outlet with 63 publications, followed by PLOS ONE (56 publications), Journal of Immunology (29), and International Immunopharmacology (30). Some leading journals with high IF were also involved, including Lancet (IF=79.323, 2 papers), Nature Medicine (IF=53.44, 2), Nature (IF=49.962, 1), Science (IF=47.728, 1), Nature Materials (IF=43.841, 1), Cell (IF=41.584, 1), and Immunity (IF=31.745, 6), manifesting potential momentousness of this topic. The dual-map overlay designed by Chen and Leydesdorff (18) could visualize the global literature proliferation at the discipline level and be used to display the interrelationship between cited and citing papers. The global base-map, which depicted the interconnection among all scientific journals in the world, divided the journals into multiple regions, which could symbolize the publishing and citation activities of various disciplines. Not only does the dual-map overlay reveal the patterns of the scientific portfolio according to the global scientific literature map, but also, additionally, is conducive to displaying the discipline concentration of relevant articles (31). In the dual-map overlay on the topic of pyroptosis in lung disease, the citing journals are on the left, and in parallel the cited journals are on the right **(** **Figure 5** **)**. Attainment in this discipline chiefly concentrates on journals related to biology, molecular science, immunology, and clinical medicine. However, utmost of the highly cited articles was issued to journals in the sectors of health, nursing, nutrition, geology, molecular, and medicine. The two thickest lines in the mapping determined primary citation pathways, implying that studies concerning molecular science, biology and genetics were exceedingly cited by the publications produced in medicine/medical/clinical and molecular/biology/immunology journals.

**Table 4:** Top 10 Journals with the most documents.

Rank	Journal	No. of documents	Total Citations	Impact factor (2020)
1	Frontiers in Immunology	63	1559	7.561
2	PLOS ONE	56	1995	3.240
3	Journal of Immunology	53	3183	5.422
4	International Immunopharmacology	47	640	4.932
5	Scientific Reports	36	962	4.380
6	International Journal of Molecular Sciences	35	661	5.924
7	American Journal of Physiology-Lung Cellular and Molecular Physiology	29	665	5.464
8	American Journal of Respiratory cell and Molecular Biology	28	704	6.914
9	Inflammation	27	502	4.092
10	Particle and Fibre Toxicology	26	1534	9.400

### Keywords

**Figure 6:**
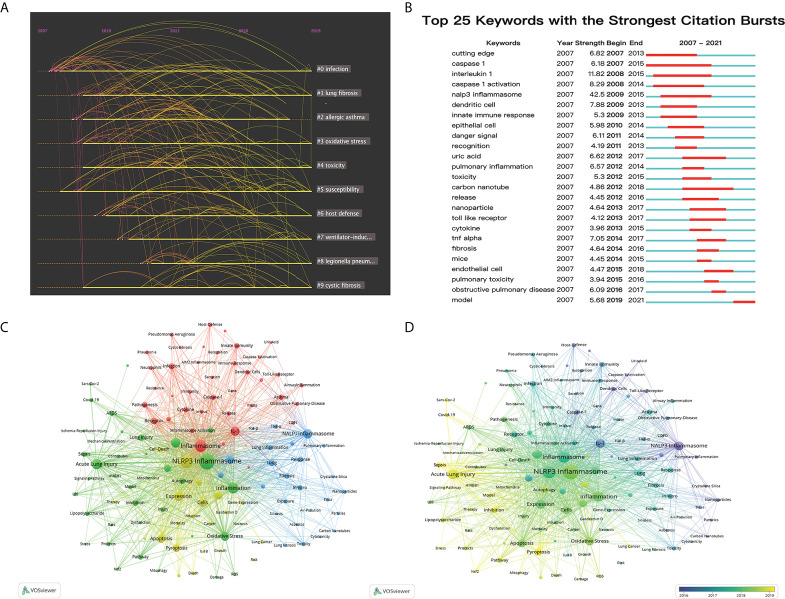
Co-occurrence keywords analysis of pyroptosis in lung disease. **(A)** The timeline diagram of the first ten clusters of keywords made by CiteSpace. **(B)** Representative burst keywords with the strongest citation bursts involved in pyroptosis in lung disease. **(C)** Visual analysis of 144 keywords that have reached the threshold of more than 20 occurrences drawn by VOSviewer. **(D)** Timeline visualization of keywords.

A total of 6412 co-occurrence keywords extracted were divided into 10 clusters by CiteSpace software **(** **Table 5** **)**. Closely related keywords were automatically classified into a cluster, and each cluster name is represented by the keyword with the largest Log-likelihood rate (LLR). The larger the LLR, the more representative the keyword is for this cluster. The modularity value (Q) of **Figure 6A** was 0.7631 and the network mean silhouette was 0.8827. Q >0.3 implies that the community structure is consequential; when the S >0.5, clustering was credible and S > 0.7 means that clustering is persuasive (32–34). The timeline graph is displayed in the cluster accordance with the year when the keyword first appears (35). The active clusters, in the early stage of research in this scientific research field, were #0 (infection), #1 (lung fibrosis), #3 (oxidative stress) and #4 (toxicity). #0 first occurred in 2007, and this was followed by a burst of studies until nowadays. #2 (allergic asthma) exhibited a burst of activity from 2008 to 2013. Subsequently, the research boom tardily declined. Since 2010, clustering #1, #2, #3, #4, #6 (host defense), #7 (ventilator-induced lung injury), #8 (legionella pneumophil) and #9 (cystic fibrosis) had been manifestly active, and the keywords of each clustering were also interrelated and studied, testifying that scientists have entered an expansive range of exploration in this sphere. Keywords with extreme citation bursts refer to the abrupt increase in a certain period, which plays a pivotal role in finding emerging topics and research subjects that have engaged much attention in a certain area (36). The top 25 keywords with the most powerful citation bursts, tending to be consequential milestones for the science mapping research, were listed in **Figure 6B**, which showed the most representative keywords in terms of burst time, burst duration, and burst strength. According to the ranking by burst time (from the past to the present), the occurrence time range of these keywords is from 2007 to 2021. ‘Cutting edge’ and ‘caspase 1’ were considerable contents that appeared in the earliest research in the field and had occupied an important position for a long time. On the one hand, ‘interleukin 1’, ‘caspase 1 activation’, ‘nalp3 inflammasome’, ‘uric acid’, ‘carbon nanotube’, being a milestone in this field for quite a long time (the length of the red line), were closely related to the research content of scholars, which marked the further excavation and discovery of research from the aspects of molecular mechanism and clinical application. On the other hand, ‘nalp3 inflammasome’ had the highest bursts strength (strength: 42.5), which implies that scholars can never ignore their equally important existence when they tend to do research in this field, follow by ‘interleukin 1’ (strength: 11.82), ‘caspase 1 activation’ (strength: 8.29) and ‘dendritic cell’ (strength: 7.88).

**Table 5:** Keyword cluster analysis of research on pyroptosis in lung disease.

Cluster ID	Size	Mean (Year)	Top terms
#0	39	2010	Infection
#1	37	2013	Lung fibrosis
#2	34	2013	Allergic asthma
#3	33	2013	Oxidative stress
#4	32	2014	Toxicity
#5	30	2015	Susceptibility
#6	30	2014	Host defense
#7	28	2015	Ventilator-induce lung injury
#8	26	2014	Legionella pneumophila
#9	25	2015	Cystic fibrosis

By importing the data into VOSviewer for co-occurrence keyword analysis, the relationship of whole keywords was visualized, and five clusters were generated through the clustering function of the software**(** **Figure 6C** **)**. Cluster 1 (yellow) was chiefly about the phenotype and mechanism of pyroptosis. Cluster 2 (blue) focused on the experimental research more pertained to the clinical implications, Cluster 3 (red) included the basic experimental content of pyroptosis in lung disease, and Cluster 4 (green) focused on clinical disease associated with pyroptosis in lung disease. There were relatively fewer keywords in Cluster 5 (purple). In addition, by 2021, the most frequently used keywords were: ‘NLRP3 inflammasome’, ‘activation’, ‘inflammation’, ‘oxidative stress’, and ‘ALI’, whose total link strength was also the maximal, which stuck out that the research on pyroptosis in lung disease mainly focused on the theme of inflammatory lung injury. The topic of pyroptosis in lung disease related to NALP3 inflammation had been perceived by scientists since 2007 **(** **Figure 6D** **)**. Two years later, NLRP3 related to pyroptosis in lung disease was first discerned by scientists in this area, but until now, it had attracted expansive attention in the field of pyroptosis in lung disease. The ‘NLRP3 inflammasome’ with the highest frequency was mainly included in #1 and #7 from the timeline diagram analyzed by CiteSpace. Likewise, it was densely correlated with the research area of lung fibrosis and ventilator-induced lung injury. The use of different colors in accordance with the average year of keywords was displayed in **Figure 6D**. ‘NALP3 Inflammasome’, ‘Caspase-1 Activation’ and ‘Dendritic Cells’ were prevailed in the early stage of research in this area. However, ‘Lung Cancer’, ‘ALI’ and ‘Nrf2’ had become a prevalent investigation object in recent years. Sars-Cov-2 (APY:2020.68), Covid-19 (APY:2020.63), and Gasdermin D (APY:2019.74) were colored yellow, manifesting that these aspects have allured increasing attention, especially in recent years, and will occupy the position of some emerging hotspots in the future.

### Co-cited reference clusters analysis

**Figure 7:**
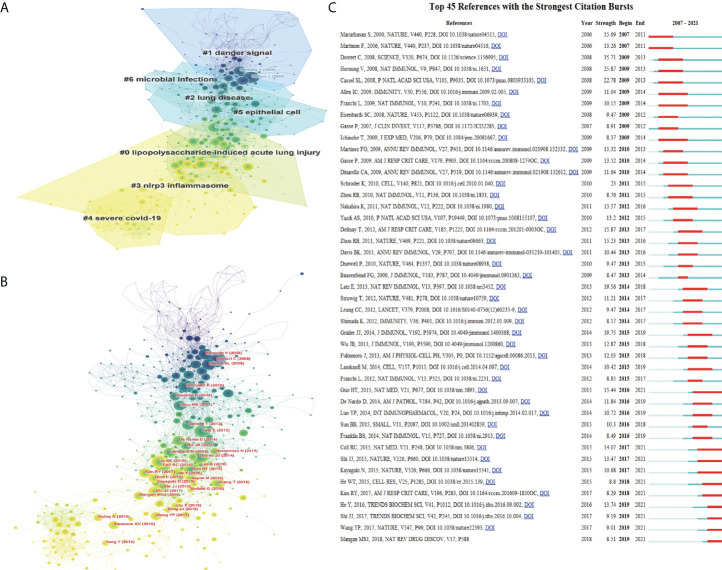
Co-cited references analysis of pyroptosis in lung disease. **(A)** The clustered network map of co-cited references related to the pyroptosis in lung disease. **(B)** Network visualization diagram of cited references. **(C)** The top 45 references with the strongest citation bursts.

A co-citation network is defined as a network of references co-cited by one or more articles at the same time. Conceptual clusters are generated when several manuscripts are cited repeatedly together (37, 38). These co-cited references were divided into 7 clusters by CiteSpace: #0 (lipopolysaccharide-induced acute lung injury), #1 (danger signal), #2 (lung disease), #3 (nlrp3 inflammasome), #4 (severe COVID-19), #5 (epithelial cell), #6 (microbial infection) **(** **Figure 7A** **)**. **Figure 7B** displayed a network visualization map of cited references, in which each node corresponded to each cited reference. The line connecting the nodes indicated the co-citation relationship. The various colors changing from purple to yellow suggested different years with a time frame of 2008 to 2021. The size of each node represents the numbers of co-citations. 141 references with the most powerful citation bursts were obtained and the top 45 among them were selected and shown in **Figure 7C**. The blue line indicates the timeline, and the intervals in which bursts were found are shown by red sections on the blue line, demonstrating the start year, the end year and the burst duration. Strongest citation burst references mean the abrupt increase of citations of certain articles in a determinate period to find emerging topics and research frontiers that have aroused much attention in the relative field (39). The paper (Strength: 35.71) with the strongest citation burstiness was a study named Innate immune activation through NALP3 inflammasome sensing of asbestos and silica published by Catherine D et al. (40) in Science in 2008. Although NALP3 had been proved to be associated with the pathological increase of IL-1β production in autoimmune syndromes (41) and inflammatory processes (42), its relationship with lung disease is not clear. They were the first scientists to consider NALP3 inflammasome into the research related to lung diseases. Their research results supported the significance of NALP3 inflammasome in lung inflammatory diseases, which were related to pathogenic air pollutants and may eventually lead to lung cancer and fibrosis. More importantly, their study provided a key basis for the ongoing exploration of NLRP3 in the treatment of lung disease.

## Discussion

In 1973, Schmeichel and Merker classified early pyroptosis into classes I, II, and III-apoptosis, autophagy-dependent cell death, and necrosis by publishing a morphological marker system (43). To provide a more accurate classification, the Nomenclature Committee on Cell Death (NCCD) put forward unified standards for the definition of cell death and different forms of cell death (44) by classifying cell death into accidental cell death (ACD) and regulated cell death (RCD) according to their functional characteristics. The innate immune system is considered to be the first line of defense against pathogens in the human body (45). It is composed of many germline-encoded receptors, which can sense invading microorganisms (46, 47). The lungs may be subject to various environmental damage, including exposure to smoke, particles, or foreign bodies in the form of pathogens such as bacteria, viruses, and fungi. This stimulation may cause airway cells such as alveolar macrophages to be damaged and initiate inflammasome mediated immune response. Chronic or acute or exposure to harmful stimulation situations, alone or in combination with susceptible genetic factors, can cause the advancement of pathological conditions (48). With these stimuli, NLR (the nucleotide-binding oligomerization domain-like receptor) began to form multi protein complexes called inflammasome. The activation of inflammasome can lead to the autoproteolytic activation of Caspase-1, which leads to the pro-inflammatory cytokine IL-1β proteolytic cleavage of precursors (30, 49, 50). Although the mechanism and function in the field of pyroptosis are not fully understood, it is an important factor in host defense when lung disease occurs (51, 52). Therefore, pyroptosis is a regulatory cell death dependent on caspase and inflammasomes. Although inflammasomes are mainly found in the maturation and secretion processes of the two important pro-inflammatory cytokines Interleukin (IL)-1β and IL-18, it has been found that they can induce RCD, and their related cell pyroptosis may be beneficial to the body’s control of pathogen replication and make it easy to phagocytize phagocytes in the next step (53). The molecular mechanism of pyroptosis mainly contains the canonical pathway of caspase-1 dependence and the non-canonical pathway, relating with caspase-4,5 (human) and caspase-11 (murine) (29). Caspase-1 is activated through a standard platform, which is assembled by pattern recognition receptors (NLRP1, NLRP3, NLRC4, AIM2 and Pyrin). However, the activation of caspase-11 is caused by the inflammatory corpuscle reaction of cells to gram-negative pathogens (8). In addition, in the non-canonical inflammasome pathway, human homologues caspase-4 and caspase-5 also lead to pyroptosis (54, 55). Caspase-1 activated by canonical typical inflammasomes, or caspase (caspase-4, -5, -11) directly recognizes bacterial lipopolysaccharide (LPS), which can specifically cut the linker between the C-terminal and N-terminal domain in gasdermin D (GSDMD), resulting in plasma membrane pore formation and pyroptosis (25). GSDMD is a candidate gene responsible for the phenotype of mouse skin mutants (56), and it is also another important component of inflammation. GSDMD is essential in the secretion of IL-1β and pyroptosis, but it does not include proteolytic matching of IL-1β. After LPS plus nigericin stimulation (4), GSDMD was recruited into NLRP3 inflammatory bodies like caspase-1. However, other gasdermin family members were not cleaved by inflammatory caspase but had a self-inhibitory effect. Some studies showed that caspase-1 wound activate caspase (caspase-3, -7) to induce apoptosis rather pyroptosis when GSDMD was absent (57). However, excessive and unnecessary pyroptosis will cause accidental injury to the body, such as chronic auto-inflammatory diseases and sepsis (58, 59).

This bibliometric analysis based on the bibliometric analysis software VOSviewer, CiteSpace, and R (4.1.1) was to make a scientific quantitative analysis of reviews and original articles written in English and published online about pyroptosis in lung disease from 2007 to the end of 2021 and displayed them through positive bibliometrics and evaluative bibliometrics. From the first time that the term “pyroptosis” was proposed in the article of scholars D’Souza and J. Heitman in 2001 to the bibliometric analysis of pyroptosis by Chinese scholars in June 2021, it can be seen that the research on pyroptosis is still in the growth stage, and the research and development in this field are worthy of scientists’ attention at present and in future (25). At the same time, this increasing trend principally depends on the plenty number of documents issued by China and the USA recently, which may be caused by the more frequent cooperation between scientists of the two countries. Therefore, not only should researchers from other countries strengthen cooperation but also jointly carry out valuable research in this sphere.

Among a total of 66 countries publishing articles on pyroptosis in lung, the largest contributor is China, followed by the USA and Germany. However, the USA has the largest number of citations as well as cited articles, and the number of citations is twice that of China. It is not the absolute relationship between the number of cited articles and citations. However, the number of citations remarkably reflects the impact of the articles in this area after they are published, which provides an important reference value for further scientific research. The number of references cited in the publications can reflect the research fundamentals and basis and provide more integral and compulsory evidence for scholars’ research results. Subsequently, they can reflect the importance and feasibility of an article and a research result from different perspectives (60). It can be seen from the radar chart of **Figure 3A** that in addition to the fewer publications than China, the USA is more prominent in the comprehensive indicators of contributions. Further speaking, the USA and Germany have paid more attention to scientific research and exploration in this area. However Chinese scholars woke up and chased closely to have made unneglectable contributions to the research development of pyroptosis in lung disease owing to the increased investment in scientific research of China in recent years. It is noticeable that more countries have cooperated closely, thus achieving a higher quantity and quality of fruition in this field, indicating that national cooperation can effectively promote high-quality research. **Figures 3E, F** delineated the close ties between countries, and Sino-American cooperation have contributed 69.70% of the total achievements in this field. Eliminating academic barriers and carrying out good academic exchanges between countries can make it possible to explore the scientific value of this field more comprehensively, quickly and accurately.

The University of California system is the organization with the largest number of papers **(** **Table 3** **)** because half of the top ten authors who have made achievements in this field are from this institution **(** **Table 2**, **Figure 4B** **)**. As shown in **Figures 4A, B**, Xia T and Wang X have established very close connections with other authors in this area, especially with the other top 10 authors. They have played a paramount role, showing that there is no national boundary in research world. Only through continuous cooperation can we enquire into a more invaluable and inconceivable world of scientific research. Xia T, currently affiliated with the University of California Los Angeles, has attained the honor of a highly cited researcher in the field of the cross-field for two consecutive years, as well as top reviewers in materials science, pharmacy and toxicology, and cross-field many times. His main exploration results focus on the content of pyroptosis in lung disease related to nanoparticle and multiwalled carbon nanotube. As early as in 1959, Richard Feynman’s celebrated speech ‘There’s Plenty of Room at the Bottom’ marked the establishment of the conceptual foundations of nanotechnology (61), and later Norio Taniguchi first proposed the term ‘nanotechnology’, which has been used until now. The achievements in amplification of nanotechnology have burgeoned in the last 15 years, but exploration on nanotechnology related to pyroptosis in lung disease did not arouse much attention until 2013 Holian A, Xia T and other prominent scientists paid special attention to this subject.

A total of 542 journals have published relevant articles, and the top ten journals account for 22.25% of the total publications. The largest number of publications belongs to Frontier of Immunology. Some journals with high IF are also thrown into to this region, illustrating the potential eminent of this topic. Not only can keywords effectively reflect the theme and research focus of an article, but also adequately help readers directly and accurately grasp the cores. Based on this point, bibliometric analysis is essential for the analysis of keywords. In the research process of pyroptosis in lung, early scientists mainly focused on the experiment and basis related to the investigation of cell biological mechanism, such as the early keywords ‘Dendritic Cells’, ‘Uric-Acid, Endothelial-Cells’ and ‘Toll-Like Receptor’. With the continuous updating and development of the scientific research system and environment, the investigation related to pyroptosis in lung disease emphasizes application of experimental and foundational studies to clinical practice, which shows that the clinical diseases related to pyroptosis in lung disease are mainly considered and provide an important way for the effective treatment of clinical diseases. The keywords in this period are ‘Necrosis’, ‘ARDS (acute respiratory distress syndrome)’, ‘COPD (chronic obstructive pulmonary disease)’, ‘Pulmonary Fibrosis’ and ‘Pneumonia’. Nowadays, with the uninterrupted improvement and expansion of exploration in this region, the keywords relationship diagram can be linked with the characteristics of people’s living environment at that time, to explore information more conducive to people’s health all over the world. The most prominent keywords at this stage are ‘SARS-Cov-2’, ‘COVID-19’, ‘Gasdermin D’, ‘Signaling Pathway’ and ‘Mitophagy’. Except to the important research objects and directions in the exploring process as shown in the **Figure 6B**, the bursts diagram also shows that the subject of pyroptosis in lung disease is primarily a breakthrough research progress and achievements in immune related aspects, such as construction of immune cell models, exploration of immune related molecules, and clinical diseases closely related to immunity. All these can prove that the current investigation in this sphere has turned to the clinical diseases or other most common clinical diseases caused by COVID-19 with high infection rates, and the study on the pathogenesis and pathway of these diseases will become the research hotspots at this stage. If keyword analysis can better grasp the frontiers and research hotspots in this field, then the analysis of co-cited literature can better understand the research status, development stages and future trends in pyroptosis in lung disease. Not only the clustering analysis of the co-cited references was obtained **(** **Figure 7A** **)**, but also **Figure 7B** reflected the citation changes and trends for each cited reference. More importantly, the top 45 references with strongest citation burst provided in **Figure 7C** were particularly worthy of reading and research by scientists involved in research in this field. Because most of them belong to articles that had produced cross-era changes and significant impact on the research of pyroptosis in lung disease, which lead to continuous exploration and breakthroughs in this area and was bound to help scientists carry out the next in-depth research and innovation. For example, Catherine D et al. introduced the NALP3 inflammasome into this field for the first time (40), the GSDMD discovered by Feng Shao’s team also had a key impact on this field (57), and Xia T et al. explored nanotechnology related to pyroptosis in lung disease (28). Their articles are all in the top 45 references with strongest citation burst.

The unknown knowledge of NLRP3, an inflammasome, has been concentrated on and explored the unknown knowledge in this research area so far. The activation of inflammasome is one of the classical mechanisms of pyroptosis, Feng Shao’s team (57) firstly found that through the activation of inflammasomes, caspase-1 can be cut to activate it and then cut GSDMD to produce N-GSDMD (62), a molecule that can punch holes in the cell membrane, activate pro-inflammatory factors, and cause pyroptosis (63). Therefore, NLRP3, extraordinarily important for inflammatory diseases, has always been a hot spot and cornerstone in this research sphere. Some studies have proved that pyroptosis is a new form of programmed cell death dependent on caspase (64), which is mainly related to NLRP3, and especially ALI is one of the most extensive inflammatory diseases in lung disease research. Currently, many professors are constantly exploring effective drugs for the efficacious treatment of ALI and ARDS. Together, the present findings demonstrated that NLRP3 inflammasome is closely related to ALI (65). In 2002, inflammasome, as a protein complex promoting inflammation, was discovered by Martin and collages (66). NLRP3 inflammasome is a protein complex of the natural immune system assembled from Caspase-1, NLRP3, the adaptor protein ASC (67). The inflammasome can instigate to the release of caspase-1-dependent pro-inflammatory cytokine IL-1β and IL-18 and then caused pyroptosis mediated by gasdermin D (68).

Lung disease is an imperative disease of the human body, with many different types of human diseases nowadays (69). Lung injury is a category of routine lung disease, especially pneumonia caused by diverse factors, the treatment of which includes radiotherapy or immunotherapy. A considerable number of studies have explored the pivotal role of pyroptosis in severe lung injury (70). In radiation-induced acute lung injury, double-stranded DNA breaks induced by ionizing radiation, can activate pyroptosis through AIM2 inflammasome (71, 72). In addition, research suggests that caspase-11-mediated endothelial pyroptosis is the basis of lung injury induced by endotoxemia (73). Not only inflammatory lung disease, but also neoplastic lung disease is also a common clinical disease. Interestingly, Wang et al. reported the anti-tumor immune function of pyroptosis through the biorthogonal system, finding that the verification of pyroptosis is helpful to enhance the anti-tumor immunity and has synergistic effect with immune checkpoint blockade (74). Yang et al. publicized that gasdermin E could trigger caspase-independent pyroptosis in cancer cells through suppressing tumor growth by granzyme B in killer cells (75). On the other hand, another study found that inflammasome played a pivotal role in reducing immune threshold, thus enhancing many autoimmune diseases (76). In 2019, severe acute respiratory syndrome coronavirus type 2 (SARS-COV-2A) infection led to the widespread spread of COVID-19 disease, which has threatened the lives of countless patients. The patient’s condition can progress from dysregulated cytokine release, pneumonia and ALI to ARDS, arrhythmia, rhabdomyolysis, shock, multisystem failure, and sorrowfully death, which directly endanger the patient’s life (77–80). Another promising finding is that inflammasome participate in severe COVID-2019 through direct infection mediated activation or indirect DAMP-mediated activation (81). Inflammasome signaling and release of IL-1β protects against a variety of pathogens, especially in the acute phase of infection (82–84).

Scientists are not only concerned about lung related pyroptosis but universally approach major diseases such as liver diseases, sepsis, cardiovascular diseases, brain diseases and central nervous system (CNS) diseases. NEK7 was found to modulate the pyroptosis of MODE-K cells by interacting with NLRP3 in an inflammatory bowel disease (IBD) model (85). Chen and his colleagues believed that NEK7 could alleviate neuroinflammation and nerve injury produced by traumatic brain injury (TBI) by affecting pyroptosis (86). Another promising finding is that the process of pyroptosis can be inhibited in human hepatocellular carcinoma (HCC) tissues and cells (87). In conclusion, pyroptosis has proved to have an imperative relationship with many lung diseases, so the in-depth study of it is the fundamental to the treatment of clinical lung diseases.

Through the auxiliary visual bibliometric analysis software, we visualized the research results of pyroptosis in the theme of lung and understood the current research situation in this field up to 2021. Nevertheless, there are still some deficiencies here. Because bibliometric analysis is closely related to timeliness, with the continuous development and exploration of researchers, the results and trends of pyroptosis in lung disease will keep pace with the times and need to be constantly updated in order to have a more comprehensive understanding and more accurate prediction of the future trend. Furthermore, some specific details cannot be ignored. For example, we only collected the articles included in SCI-expanded of WoSCC, and there are some other research findings that may not be included in the database. Secondly, we only collected the articles in English. For more comprehensive bibliometric analysis in the future, we can collect them in multiple languages. Nevertheless, this visualization method to understand the research status, hot spots, and development trends in a certain field is still worth using. It can help scholars understand relevant content more effectively and quickly and explore further expeditiously.

## Conclusion

Through CiteSpace, VOSviewer software and R software, we have carried out a bibliometric analysis on pyroptosis in pyroptosis in lung disease and analyzed and visualized the two different types of indicators through positive bibliometrics and evaluative bibliometrics. The research in this field shows an upward trend year by year. Among them, China enjoys the largest number of documents, and the USA enjoys the largest number of citations. The USA has paid constant attention to this field, and China is a rising star in many institutions. Half of the leading institutions belong to China, and most of the authors who make major contributions in this field belong to the US institutions when publishing articles. Frontier of Immunology is the magazine with the largest number of articles in this field. Many influential international journals have published articles in this field, which shows that the role of pyroptosis in lung disease has important research value and broad application prospects. At present, the research hotspots in this field lie in NLRP3, inflammasome, and ALI, with the focus on its related mechanism and clinical therapeutic potential. It should be noted that bibliometrics is a scientific research method closely related to timeliness. For the understanding and exploration of a field, we should closely track the real-time scientific research results in order to make a more accurate understanding and exploration of unknown fields.

## Data availability statement

The raw data supporting the conclusions of this article will be made available by the authors, without undue reservation.

## Author contributions

WM, QL and JW conceived the study. WM, HZ, XS, HY were involved in the data collection and analysis. WM, QL and ZX wrote the manuscript. HZ and XL revised the manuscript. All authors contributed to the article and approved the submitted version. WM, QL and HZ contributed equally to this work.

## Funding

This study was supported by grants from the National Natural Science Foundation of China (Grant No. 81871601); The Clinical Research Special Project of Shanghai Municipal Health Commission (No. 202040004); Natural Science Foundation of Shanghai (No. 22ZR1452200); Program of Shanghai Academic Research Leader (No.21XD1402800); Shanghai “Rising Stars of Medical Talent” Youth Development Program: Outstanding Youth Medical Talents.

## Conflict of interest

The authors declare that the research was conducted in the absence of any commercial or financial relationships that could be construed as a potential conflict of interest.

## Publisher’s note

All claims expressed in this article are solely those of the authors and do not necessarily represent those of their affiliated organizations, or those of the publisher, the editors and the reviewers. Any product that may be evaluated in this article, or claim that may be made by its manufacturer, is not guaranteed or endorsed by the publisher.
